# Supplementation of *Aspergillus oryzae* Culture Improved the Feed Dry Matter Digestibility and the Energy Supply of Total Volatile Fatty Acid Concentrations in the Rumen of *Hu* Sheep

**DOI:** 10.3389/fnut.2022.847156

**Published:** 2022-04-25

**Authors:** Long Guo, Duihong Zhang, Ruifang Du, Fadi Li, Fei Li, Tao Ran

**Affiliations:** State Key Laboratory of Grassland Agro-Ecosystem, Key Laboratory of Grassland Livestock Industry Innovation, Ministry of Agriculture and Rural Affairs, Engineering Research Center of Grassland Industry, Ministry of Education, College of Pastoral Agriculture Science and Technology, Lanzhou University, Lanzhou, China

**Keywords:** nutrient digestibility, sheep, *Kiritimatiellaeota*, rumen bacterial community, *Aspergillus oryzae* culture

## Abstract

The objective of the present study was to investigate the effects of feeding different amounts of *Aspergillus oryzae* culture (AOC) on the degradation rate of various feeds for 24 h, rumen fermentation parameters, microbial community, and blood cell composition of *Hu* sheep. Sixteen castrated and fattening adult *Hu* sheep with permanent rumen fistula were randomly divided into four groups (four sheep per group) based on body weight (64.62 ± 5.83 kg). The experiment was repeated for two periods to ensure eight replicates for each treatment, and each period consisted of 28 days, including a 7-d of transition, a 14-d of pre-feeding, and a 7-d of sample collection. The control group (CON) received a basal diet without AOC, and the other groups were fed basal diet supplemented with 10 g/d, 20 g/d, and 40 g/d AOC, respectively, every day before the morning feeding. Supplementation with 20 g/d and 40 g/d AOC significantly increased (*P* < 0.05) the total volatile fatty acids (TVFAs) content, the molar ratio of butyric acid, and the 24 h dry matter (DM) degradation rate of alfalfa hay and corn straw. When fed 40 g/d AOC, the DM degradation rate of corn germ meal and the relative abundance of *Kiritimatiellaeota* were significantly increased (*P* < 0.05), but the ratio of acetic acid to propionic acid (A/P) was significantly reduced (*P* = 0.04). In conclusion, supplementation with AOC for *Hu* sheep could improve feed DM digestibility and increase the energy supply of TVFAs concentration in the rumen. Based on the feed conditions of the present study, supplementation 40 g/d of AOC could increase the production efficiency of sheep while higher level have to further investigate.

## Introduction

Antibiotics have been widely used since the 1940s to build the immunocompetence of livestock against infectious diseases and as growth promoters (GPs). However, long term use of in-feed antibiotics as GPs will lead to the development of drug resistance bacteria, which can further be transferred to humans and be potential threatens to human health (1). With increasing concerns toward the use of antibiotics in the ruminant feed industry, more emphasis has been given to increase public awareness on the issue, disease prevention, and the use of other natural GPs, such as direct-fed microbials (DFMs) (2).

DFMs are mono or mixed cultures of live microbes that exert beneficial health effects by improving gastrointestinal tract microbial balance when fed to the host (3). *Aspergillus oryzae* culture (AOC) is one of the most common DFM product and has been widely used as a feed additive in ruminant production (4–6). Studies have shown that AOC can regulate rumen microbiota (7), improve fiber digestibility (8). Supplementation of *Aspergillus oryzae* (AO) in diets of lactating cows increased milk production, feed efficiency, and tolerance to heat stress in some (9) but not all (10, 11) studies. However, reports about the effects of AOC on the rumen microbial community and characteristics of sheep are relatively scarce. The effective dosage of AOC in the production of ruminants such as sheep has not yet been reported. Most of the additive doses reported in existing studies are less than 10g, and the effects were different. As a nutritional additive, AOC is an excellent protein raw material. Therefore, increasing the addition amount in production may produce better results.

The rumen microbiome includes various microbes, such as bacteria, fungi, protozoa, archaea, and bacteriophages. The rumen microbiome plays a crucial role in shaping digestion physiology and ruminant production, and its potential utility for health and performance manipulation (12). A key reason for this is that rumen microbes could help ruminants transform fibrous and nonfibrous plant material into meat and milk. Therefore, regulating ruminal microbes and fermentation could improve feed efficiency in ruminants, that means a lot especially as the costs of forage and cereals have been continuously increased in recent years.

In the sheep industry, the sheep should be fed by balanced diets which contain all kinds of nutrients even some small feed additives to improve nutrients digestion and absorption, promote ruminal fermentation and hematological parameters, ultimately enhance the growth performance and productivity of sheep (13). Hematological indicators could reflect the health status of animals. To assess the impact of additives such as AOC on sheep health, hematological indicators may be timelier and more effective. Nowadays, *Hu* sheep husbandry has gradually become large-scale and intensive production (14), which has brought about some new problems, especially the high incidences of nutritional and metabolic diseases. The disorder of rumen microbiota is one of the causes of these diseases. Therefore, the use of DFMs to stabilize the rumen environment while ensuring the efficient operation of the digestive capacity of the gastrointestinal tract is being considered as the best strategy to solve the problem of antibiotics being disabled.

The DFMs feed additives with dual characteristics of nutritional and non-nutritional have improved the utilization rate of nutrition digestion and regulated the health of animals (15), especially AOC. However, whether AOC could increase the rumen digestibility, shape rumen microbiome, improve the health status of *Hu* sheep have not been reported. Here, the objective of the present study was to investigate the effects of supplementation with different amounts of AOC on the degradation rate of various feeds for 24 h, rumen fermentation parameters, microbial community, and blood cell composition of *Hu* sheep.

## Materials and Methods

All experimental protocols were approved by the animal protection committee of Gansu Province, China (2005-12), and the experimental procedures used in this study were in line with the University's animal research guidelines.

### Animals, Diets, and Experimental Design

Sixteen castration adult *Hu* sheep with permanent rumen fistulas were blocked by body weight (64.62 ± 5.83 kg) and randomly assigned to one of the four treatments. The surgical operation of rumen fistulas adopts one-step installation method by using T-shaped fistula. The post-operative care procedure referred to sheep fistula surgery concepts and techniques from Durmic et al. (16). The details of the rumen operation and sheep care were described in recently publication by our research team (17). The treatments were control group (CON) that fed a basal diet in the form of total mixed ratio (TMR), and experimental groups fed a basal diet plus 10, 20 and 40 g of AOC/d, respectively. Each treatment had eight replicates as the experiment was repeated for two periods, with each period consisted of a 7-d of transition, a 14-d of pre-feeding and, a 7-d for sample collection. All sheep were housed in a single stall (1.4 × 0.75 × 1.5 m) that equipped with trough and tank. All sheep were fed twice daily at 8:00 and 18:00 with TMR. The experimental treatments feed different amount of AOC before morning feeding. *In situ* degradation of feeds includes roughage (corn straw and alfalfa hay), corn, and corn germ meal. The compositions and nutrient levels of the basal diet are shown in Table 1. The AOC was composed of AO soybean meal culture and microbial metabolites. The AOC in this study was provided by Haiyi Biological Feed Co., Ltd. (Guangdong, China). The nutrient composition and enzyme activity of the AOC (all measured values on a dry matter basis) are shown in Table 2.

**Table 1:** Composition and nutrient levels of the basal experimental diets (DM basis).

**Items**	**Contents**
**Ingredients**	
Corn stalk, %	20.0
Corn, %	30.0
Concentrate supplement, %	47.3
Limestone, %	1.2
NaCl, %	0.5
Permix^*a*^, %	1.0
Total, %	100.0
**Nutrient levels** ^***b***^ **, %**	
DM, %	95.70
OM, %	91.49
CP, %	13.41
EE, %	2.98
NDF, %	39.21
ADF, %	22.61
GE, MJ/kg	17.07

a *The premix each kilogram of diet is provided: Fe 25 mg, Mn 40 mg, Zn 40 mg, Cu 8 mg, I 0.3 mg, Se 0.2 mg, Co 0.1 mg, AVA 940 IU, DVD 111 IU, EVE 20 IU*.

b *Nutrients and gross energy were measured*.

*DM, dry matter; OM, organic matter; CP, crude protein; EE, ether extract; NDF, neutral detergent fiber; ADF, acid detergent fiber; GE, gross energy*.

**Table 2:** Nutrients and active components of *Aspergillus oryzae* culture (DM basis).

**Items**	**Contents**
DM, %	93.47
OM, %	86.60
CP, %	52.91
EE, %	1.37
NDF, %	11.16
ADF, %	7.73
GE, MJ/kg	19.18
AO, hundred million/g	≥2
Neutral protease activity^*a*^,IU/g	1020
Alkaline protease activity^*a*^,IU/g	770
Cellulase activity^*a*^,IU/g	482

a *The active components come from the product description*.

*DM, dry matter; OM, organic matter; CP, crude protein; EE, ether extract; NDF, neutral detergent fiber; ADF, acid detergent fiber; GE, gross energy, AO, aspergillus oryzae*.

### Sample Collection

Rumen contents were collected through rumen cannulas before and after feeding (0, 2, 4, 6, 8, and 12 h) on d 19 to 20 of each experimental period. Collected rumen contents were mixed evenly, filtrated through four layers of gauze, with the pH measured immediately using a portable pH meter (Leimin PHSJ-4A desktop acidity meter, Shanghai Leimin Sensor Technology Co., Ltd.). Five milliliters of filtrates were collected into a 10 mL cryopreserving tube that containing 1 mL of metaphosphoric acid (25% wt/vol), stored at−80°C for volatile fatty acid (VFA) extraction. Rumen contents before morning feeding were collected and stored in a 50 mL cryopreservation tube at−80°C for microbial DNA extraction. Blood samples were collected from the jugular vein before morning feeding (08:00 am) on the 28th day of each period.

### Measurement of Dynamic Rumen pH

From d 15 to 18 of each experiment period, the ruminal pH was continuously monitored for 3 days using a dynamic pH continuous monitoring and recording system (IP-600-10, JENCO, Shanghai, China). The pH electrode was calibrated with pH 4.00 and 6.86 standard fluids, placed in the ventral sac of the rumen through the rumen fistula before morning feeding (8:00 am) on d 15 and taken out on d 18, with ruminal pH data recorded every 0.5 min.

### The *In-situ* Incubation

*In situ* incubation of substrates including corn straw, alfalfa hay, corn, and corn germ meal was conducted on d 27 to 28 of each period according to previous studies (18, 19). Four grams (accurate to 0.0001 g) of corn straw, alfalfa hay, corn, and corn germ meal that grounded to pass through a 2-mm screen were weighed into pre-numbered and dried nylon bags with constant weight (size 5 × 7 cm, pore 300 mesh). The prepared nylon bags were placed into the rumen of each sheep just before morning feeding, and ensure immersion in rumen abdominal bursal digest. After 24 h of incubation, all nylon bags were removed from the rumen, immediately submerged in ice water to stop microbial activity, rinsed with running water until the water was clear. The bags were dried in a constant temperature drying oven at 65°C for 72 h, colled in a desiccator, and weighed. Weights from each bag was used to calculate DM disappearance rage in the rumen (20). Unincubated bags (0 h) were washed and dried along with the incubated bags to calibrate the soluble fraction.

### Rumen Fermentation

The method of extraction and determination of VFA was according to Li et al. (21). Rumen fluid were thawed at 4°C and centrifuged at 2,500 × g for 5 min. Five milliliters of supernatant were taken into a centrifuge tube containing 1 mL 25% metaphosphoric acid. It was left for 3 h at 4°C and centrifuged at 3000 g for 10 min. Then, two milliliters supernatant at 12,000 × g at 4°C for 15 min, filtered with 0.45 μm organic membrane, and 1 mL of the filtrate was extracted and mixed with 200 μL 1% crotonic acid. The VFA content in rumen fluid was determined by a gas chromatograph (GC-2010 PLUS, Shimadzu, Japan).

### Blood Cell Counts

An automatic blood cell analyzer (IDEXXPro CyteDx TM, Sysmex Corporation, Kobe, Japan) was used to determine white cell count (WBC), red blood cell content (RBC), neutrophil count (NEUT), lymphocyte count (LYMPH), mononucleosis (MONO), eosinophilia (EO), basophilic (BASO), hemoglobin (HGB), hematocrit (HCT), mean corpuscular volume (MCV), mean corpuscular hemoglobin (MCH), mean corpuscular hemoglobin concentration (MCHC), platelet count (PLT), mean platelet volume (MPV), and platelet hematocrit (PCT).

### Rumen Bacterial Community

Based on the results of the DM degradation and rumen fermentation, the CON and supplementation of 40 g/d AOC treatments were selected to measure the 16S rDNA abundance in rumen contents. Microbial DNA was extracted according to the method of Yu and Morrison (22), and the obtained samples (n = 16) were sequenced using the platform of Illumina Hiseq 2500 (BaiMike Biotechnology Co., Ltd., Beijing, China) for 16S rDNA analysis. By PCR amplification, purification, quantification, and homogenization, a sequencing library (SMRT Bell) was formed. The CCS sequence was obtained using the Smart Link tool provided by PacBio. Limav1.7.0 software was used to identify CCS through barcodes and obtain RAW-CCS sequence data. Cutadapt1.9.1 software was used to identify and remove primer sequences and conduct length filtering to obtain clean-CCS sequences without primer sequences. Finally, chimeras were removed (UCHIMEv4.2 software), and the effective-CCS sequence was obtained. The high-quality sequences were clustered and divided by OTUs with a 97% sequence similarity. Based on OTUs, the samples were analyzed by taxonomy, and original data and community structure maps were obtained at the phylum, class, order, family, and genus levels. The ACE, Chao1, Shannon and, Simpson indices were obtained, and principal coordinates analysis (PCoA), principal component analysis (PCA), and nonmetric multidimensional scaling (NMDS) were performed to compare species diversity and richness among different samples.

### Statistical Analysis

Statistical analyses were performed using Statistical Analysis System 9.4 software (SAS, Inst. Inc. Cary, NC). The GLM procedure was used to conduct a one-way analysis of variance for the experiment results. The model of statistics was as follows: Y_i_ = μ + A_i_ + B_i_, in which Y_i_, μ, A_i_, and B_i_ represented the dependent variable, overall mean, diet effect, and error term, respectively. Duncan's test was performed to analyze the multiple comparisons of means. VFA data were analyzed by a two-factor ANOVA with different feeding amounts and sampling time points as factors. The model of statistics was as follows: Y_ijk_ = μ_T_ + A_i_ + B_i_ + R_ij_ + e_ijk_, in which Y_i_, μT, A_i_, B_i_, R_ij_, and e_ijk_, represented the dependent variable, overall mean, diet effect, time effect, and error term, respectively. The microbial diversities were analyzed by an independent two-sample *T*-test. *P* < 0.05 indicates a significant difference, *P* ≥ 0.05 indicates no significant difference, and 0.05 < *P* < 0.10 indicates a significant tendency.

## Results

### Degradation Rate of Dry Matter in the Rumen for 24 h

The DM degradation rate at 24 h of alfalfa hay, corn straw, corn, and corn germ meal increased linearly with increasing AOC supplementation amount (*P* < 0.05, Table 3). When supplemented with 20 g/d and 40 g/d AOC, the DM degradation rate of alfalfa hay and corn stalk was significantly higher than that of CON (*P* < 0.05). The DM degradation rate of corn germ meal when fed 40 g/d AOC was significantly higher than that of CON (*P* = 0.006).

**Table 3:** Effects of different dietary levels of *Aspergillus oryzae* culture supplementation on the DM disappearance of different feed materials.

**Items**	**Treatments**				**SEM**	***P*** **-value**	
	**CON**	**AOC10**	**AOC20**	**AOC40**		**Linear**	**Quadric**
Alfalfa hay, %	44.79^c^	47.64^bc^	50.04^ab^	53.09^a^	0.686	<0.001	0.852
Corn stalk, %	22.84^c^	24.72^bc^	26.97^ab^	26.97^a^	0.738	0.001	0.593
Corn, %	51.43	52.37	54.42	57.93	1.118	0.033	0.902
Corn germ meal, %	33.95^b^	36.16^ab^	38.56^ab^	41.38^a^	1.015	0.006	0.712

a, b, c *Within a row, means with different letters differ (P < 0.05)*.

*CON, AOC10, AOC20, AOC40 are represent supplementation with Aspergillus oryzae culture 0 g/d, 10 g/d, 20 g/d, and 40 g/d, respectively*.

### Dynamic Parameters of Rumen pH

**Figure 1:**
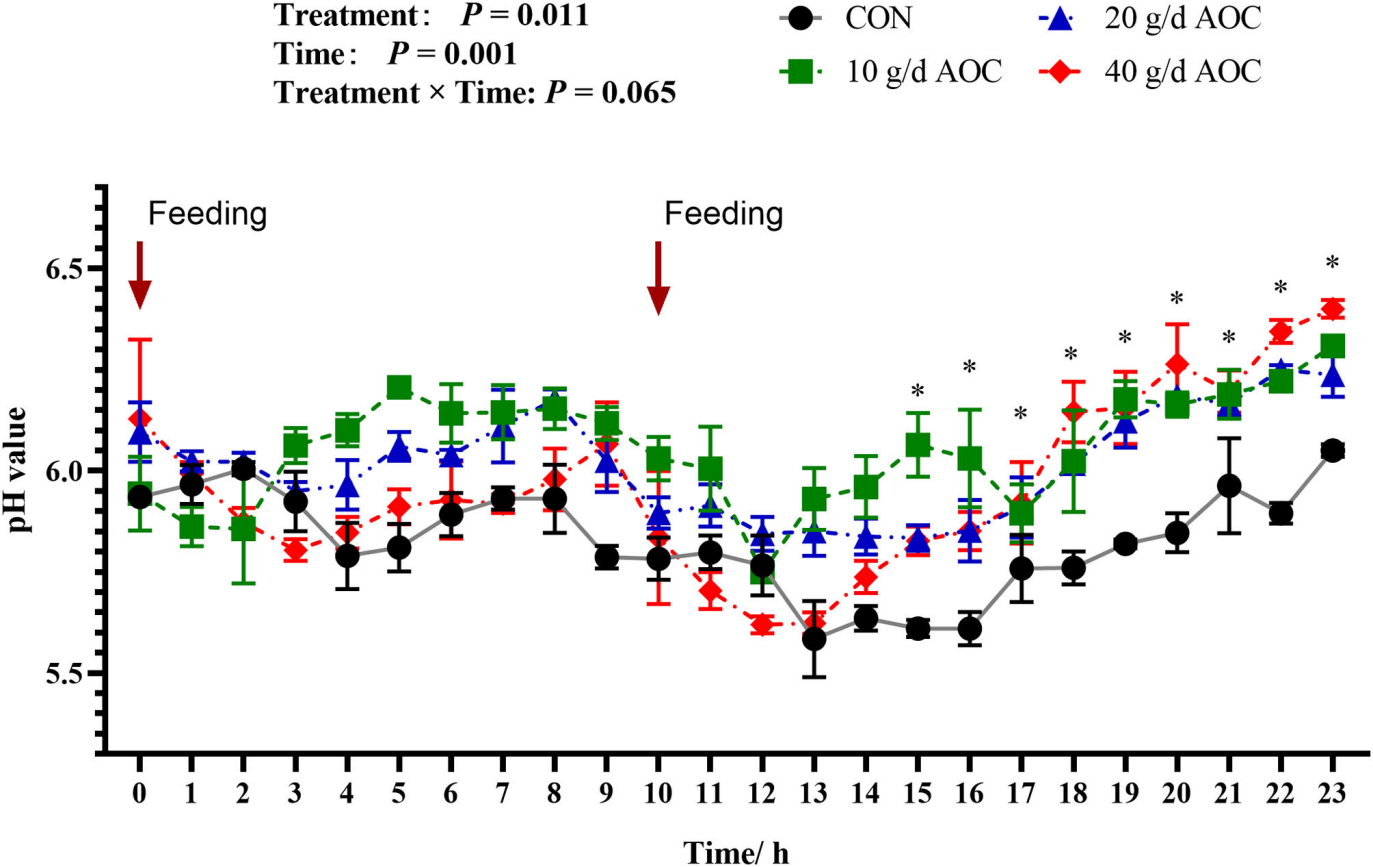
Dynamic changes of ruminal pH of Hu sheep supplemented with different levels of *Aspergillus oryzae* culture. The arrows indicate the feeding time.

The dynamic variation pattern of rumen pH over 24 h was shown in Figure 1. The global pH of all treatments was below to 7.0. When the morning feeding started, the pH of the CON and AOC-10g/d supplementation group dropped rapidly to below 6.0. As digestion progressed, the overall pH decreased first and then increased. When the second feeding started, the pH of the CON group dropped rapidly and continued to the minimum value of 5.5. During the digestion process, the pH values of the AOC treatment groups were significantly higher than that of the CON group after a second feeding for 5 hours and lasted until the end of the process. Overall, rumen pH fluctuated to different degrees before and after feeding; it decreased earlier and then recovered.

### Rumen Fermentation

According to Table 4, VFA contents were not significantly different at different time points and treatments. Principal component analysis showed that rumen total VFA content and the molar ratio of butyric acid were significantly higher than those in other groups (*P* < 0.05) when the supplementation was 20 g/d and 40 g/d AOC. The isovalerate molar ratio was decreased with 20 g/d and 40 g/d AOC supplementation (*P* < 0.05). The minimum value of the ratio of acetate to propionate was shown in the AOC-40 g/d treatment group (*P* < 0.05). Valerate acid contents in the AOC-40 g/d group was lower than that of the other groups (*P* = 0.031). The molar ratios of acetic acid, propionic acid, and isobutyric acid were not significantly different among treatments (*P* > 0.05). The content of total VFAs and the molar ratio of propionic acid after feeding were significantly higher than before feeding (0 h) (*P* < 0.05). There were no significant differences in acetic acid, butyric acid, or valerate content at any time point (*P* > 0.05). Isobutyric acid, isovalerate, and the ratio of A/P decreased after feeding (*P* < 0.05). Isobutyric acid contents at 2 h and 12 h were significantly greater than those at 4 h, 6 h, and 8 h (*P* < 0.001). Isovalerate contents were lowest at 4 h and highest at 12 h (*P* < 0.001). The ratio of A/P significantly decreased after feeding (*P* = 0.04). There was no interaction between different treatments and VFA contents at any time point (*P* > 0.05).

**Table 4:** Effects of different dietary levels of *Aspergillus oryzae* culture supplementation on ruminal VFA composition of *Hu* sheep.

**Items**	**Treatments**				**Time^1^**						***P*** **-value**		
	**CON**	**AOC10**	**AOC20**	**AOC40**	**0**	**2**	**4**	**6**	**8**	**12**	**Treatment**	**Time**	**Interaction**
TVFA (mmol/L)	124.35^b^	125.65^b^	148.55^a^	146.73^a^	114.86^b^	135.12^a^	141.05^a^	141.59^a^	140.77^a^	144.53a	<0.001	0.003	0.417
Acetate (%)	49.15	45.4	49.08	47.31	44.63	46.49	47.89	49.13	47.89	50.46	0.187	0.216	0.981
Propionate (%)	32.81	31.5	34.19	35.69	25.13b	33.50a	35.64a	35.53a	35.70a	35.52a	0.216	<0.001	0.98
Isobutyrate (%)	0.86	0.87	0.79	0.7	1.31^c^	0.86^a^	0.62^b^	0.65^b^	0.63^b^	0.83^a^	0.124	<0.001	0.835
Butyrate (%)	23.60^b^	23.73^b^	25.72^a^	25.10^a^	22.44	25.05	23.36	24.04	22.96	24.8	<0.001	0.445	0.942
Isovalerate (%)	1.78^a^	1.82^a^	1.61^ab^	1.32^b^	2.35^a^	1.63^ab^	1.31^c^	1.38^ab^	1.44ab	1.75a	0.01	<0.001	0.969
Valerate (%)	2.39^a^	2.08^b^	2.34^a^	2.35^a^	2.2	2.39	2.14	2.27	2.29	2.45	0.031	0.276	0.997
A/P	1.53^a^	1.53^a^	1.49^ab^	1.36^b^	1.77^a^	1.44^b^	1.37^b^	1.45^b^	1.37^b^	1.48^b^	0.04	<0.001	0.723

a, b, c *Within a row, means with different letters differ (P < 0.05)*.

1 *0, 2, 4, 6, 8, 12 indicate that rumen fluid collection time is before feeding, and 2, 4, 6, 8, 12 h after feeding, respectively*.

*CON, AOC10, AOC20, AOC40 are represent supplementation with Aspergillus oryzae culture 0 g/d, 10 g/d, 20 g/d, and 40 g/d, respectively; A/P, acetic acid to propionic acid ratio*.

### Rumen Bacterial Community

Alpha diversity indices, including the ACE, Chao1, Simpson, and Shannon, of the samples in the CON and AOC-40 g/d group were shown in Table 5. The OUTs, ACE, Simpson, and Shannon were not significantly different (*P* > 0.05) between these two groups.

**Table 5:** Effects of *Aspergillus oryza*e culture supplementation on α diversity indices of rumen microbial community of *Hu* sheep.

**Items**	**Treatments**		**SEM**	***P*-value**
	**CON**	**AOC40**		
OTU	297	339	21.47	0.347
ACE	392	404	22.66	0.794
Chao1	400	406	22.91	0.901
Simpson	0.92	0.95	0.022	0.562
Shannon	5.83	6.18	0.267	0.526
Coverage	0.97	0.98	0.003	0.055

*CON and AOC40 are represent supplementation with Aspergillus oryzae culture 0 g/d and 40 g/d, respectively*.

*OUT, operational taxonomic units; ACE, the ACE estimator*.

At the phylum level, *Bacteroidetes* and *Firmicutes* were the dominant phyla, representing averages of 45.21%, 42.99%, and 34,96%, 37.10% in the two treatments, respectively (Table 6). Compared with CON, the AOC-40 g/d treatment significantly increased the abundance of *Kiritimatiellaeota* in the rumen (*P* = 0.017, Table 6). There were no significant differences in the abundance of *Actinobacteria, Firmicutes, Patescibacteria*, or *Bacteroidetes* between treatment groups (*P* > 0.05). At the genus level, there was no significant difference between the two groups (Table 7).

**Table 6:** Effects of *Aspergillus oryzae* culture supplementation on the phylum-level average relative abundance (% of total reads) of rumen microbial community of *Hu* sheep.

**Items**	**Treatments**		**SEM**	***P*-value**
	**CON**	**AOC40**		
*Bacteroidetes*	45.21	42.99	1.909	0.582
*Firmicutes*	34.96	37.10	2.420	0.675
*Proteobacteria*	9.35	5.04	1.606	0.190
*Tenericutes*	2.65	2.04	0.306	0.382
*Spirochaetota*	2.32	2.19	0.268	0.821
*Kiritimatiellaeota*	1.87^a^	5.37^b^	0.774	0.017
*Fibrobacteres*	1.28	1.81	0.411	0.543
*Patescibacteria*	0.76	0.86	0.149	0.747
*Actinobacteria*	0.02	0.09	0.021	0.103

a, b *Within a row, means with different letters differ (P < 0.05)*.

*CON and AOC40 are represent supplementation with Aspergillus oryzae culture 0 g/d and 40 g/d, respectively*.

**Table 7:** Effects of *Aspergillus oryzae* culture supplementation on the genus-level average relative abundance (% of total reads) of bacteria genus of rumen microbial community of *Hu* sheep (%).

**Items**	**Treatments**		**SEM**	***P*-value**
	**CON**	**AOC40**		
*Prevotella*_1	26.86	28.33	2.350	0.767
*Ruminococcus*	6.79	6.31	1.069	0.833
*Prevotellaceae*_UCG-001	3.67	2.93	0.746	0.640
*Succiniclasticum*	2.74	1.93	0.444	0.378
*Treponema*_2	2.32	2.18	0.268	0.811
*Christensenellaceae*_R-7_group	4.68	5.66	1.176	0.693
*Rikenellaceae*_RC9_gut_group	2.91	2.11	0.299	0.187
*Lachnospiraceae*_XPB1014_group	2.22	2.15	0.398	0.929
*Ruminococcaceae*_NK4A214_group	2.61	3.23	0.425	0.484
*Fibrobacte*r	1.35	2.00	0.425	0.479
*Ruminococcaceae*_UCG-014	1.25	1.38	0.203	0.771
*Veillonellaceae*_UCG-001	0.84	0.88	0.159	0.893
*Lachnospiraceae*_NK3A20_group	0.99	1.44	0.213	0.301

*CON and AOC40 are represent supplementation with Aspergillus oryzae culture 0 g/d and 40 g/d, respectively*.

**Figure 2:**
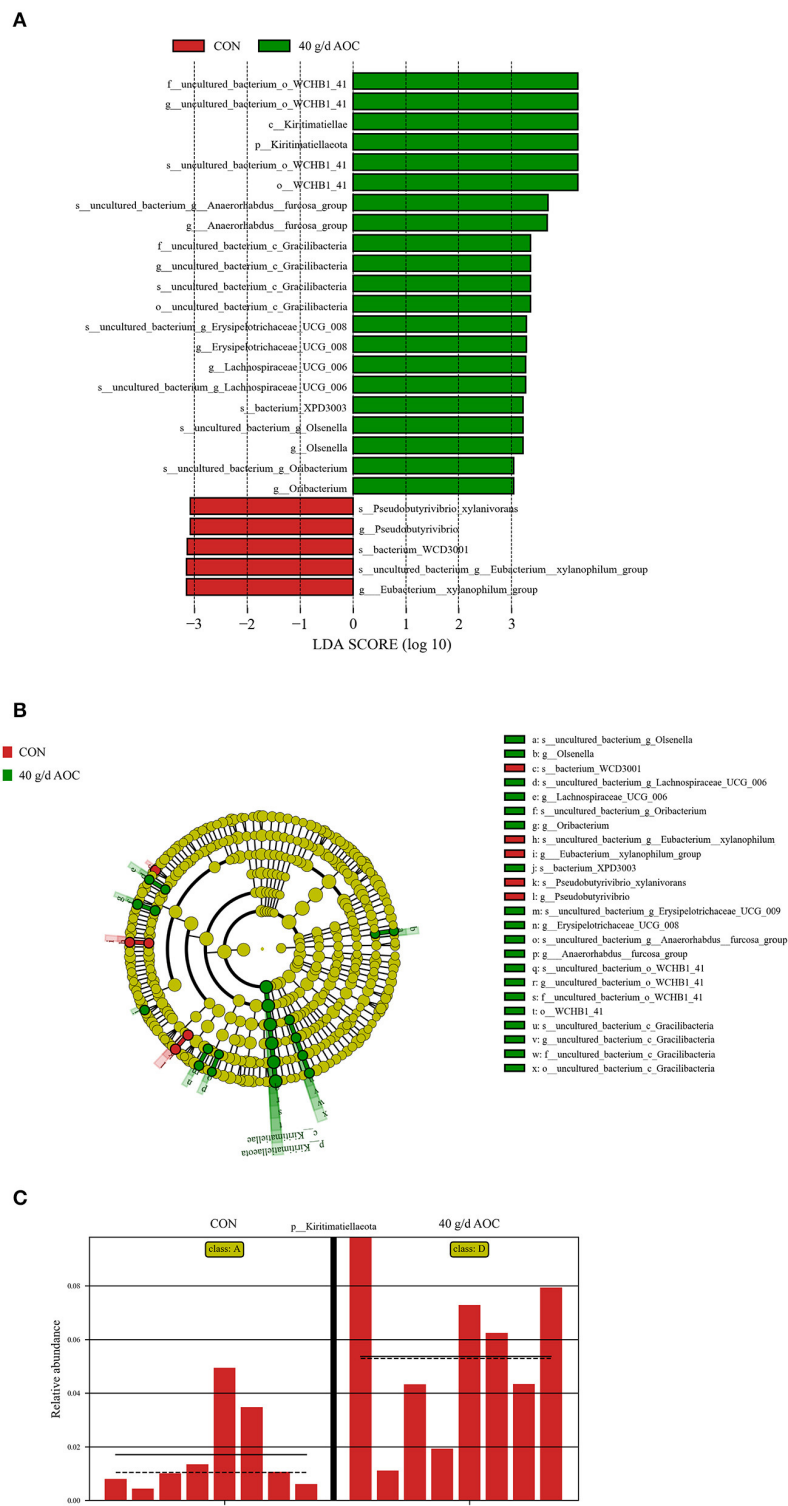
Histogram of the line discriminant analysis (LDA) effect size **(A)**, cladogram **(B)**, and relative abundance of *Kiritimatiellae* **(C)** in the 40 g/d AOC group compared with CON. Only the genera with a linear discriminant analysis significant threshold > 2 are shown.

The results of the line discriminant analysis (LDA) effect size (Figure 2A) showed that 26 genera were identified as discriminative features between the samples taken from the CON and AOC-40 g/d supplementation groups. Five genera were enriched in the CON group. Twenty-six genera were significantly enriched in the AOC-40 g/d group (Figure 2B), of which *Kiritimatiellaeota, Kiritimatiellae*, and some types of WCHB1_41 s were the top most enriched. The relative abundance of *Kiritimatiellaeota* was significantly increased in the AOC-40 g/d group (Figure 2C).

**Figure 3:**
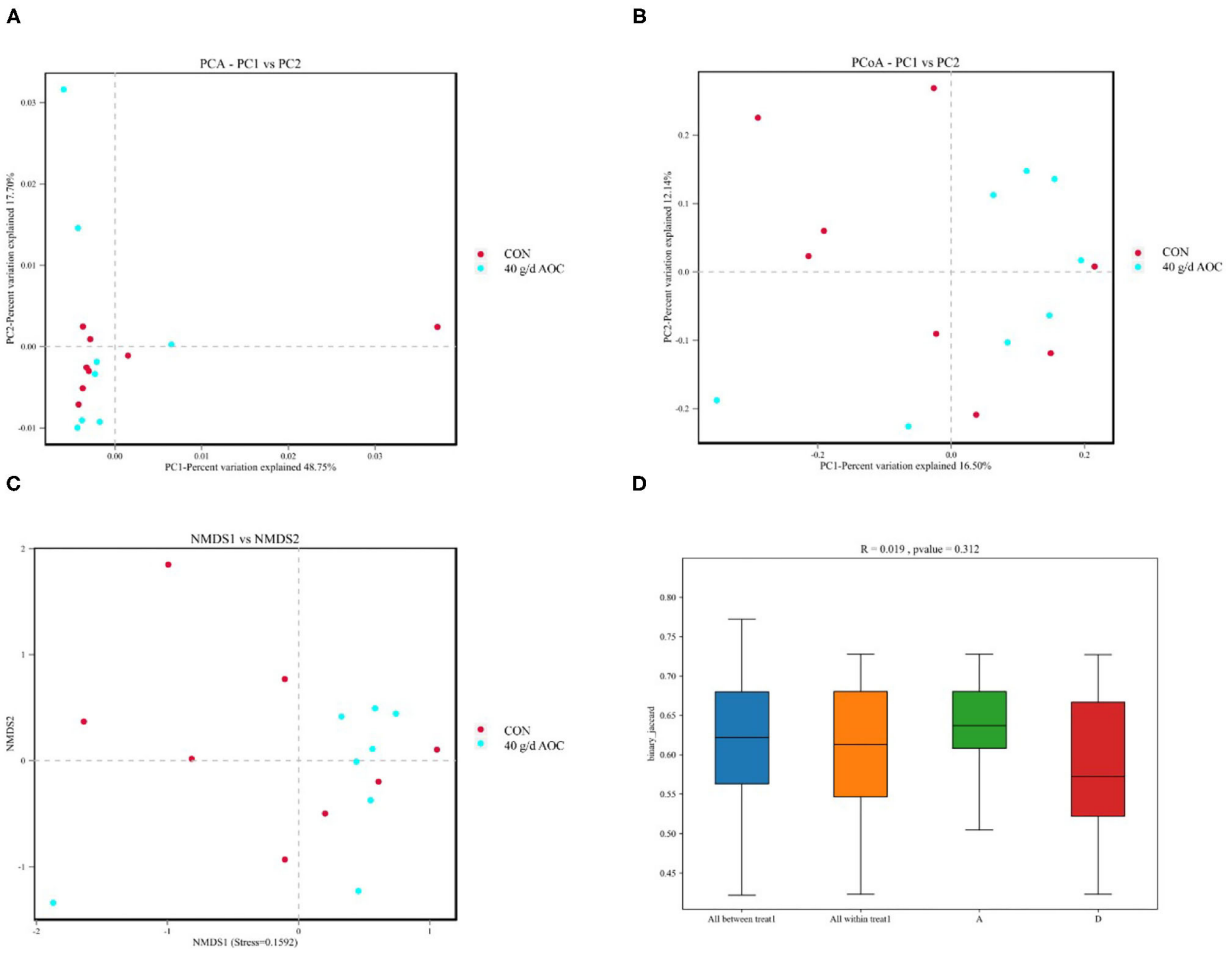
Beta diversity analysis of *Aspergillus oryzae* culture supplementation on rumen microbial community of *Hu* sheep. Points represent each sample; different colors represent different groups. **(A)** PCA, Principal component analysis; **(B)** PCoA, Principal coordinates analysis; **(C)** NMDS, Nonmetric multi-dimensional scaling; **(D)** Permanova (Adonis) analysis.

The differences in rumen microbiota between the CON and AOC-40 g/d groups were further analyzed using β-analysis, including principal component analysis (PCA, Figure 3A), principal coordinate analysis (PCoA, Figure 3B), nonmetric multidimensional scaling (NMDS, Figure 3C), and permanov analysis (Figure 3D), and the results showed that the two sets of samples were not clearly separated.

**Figure 4:**
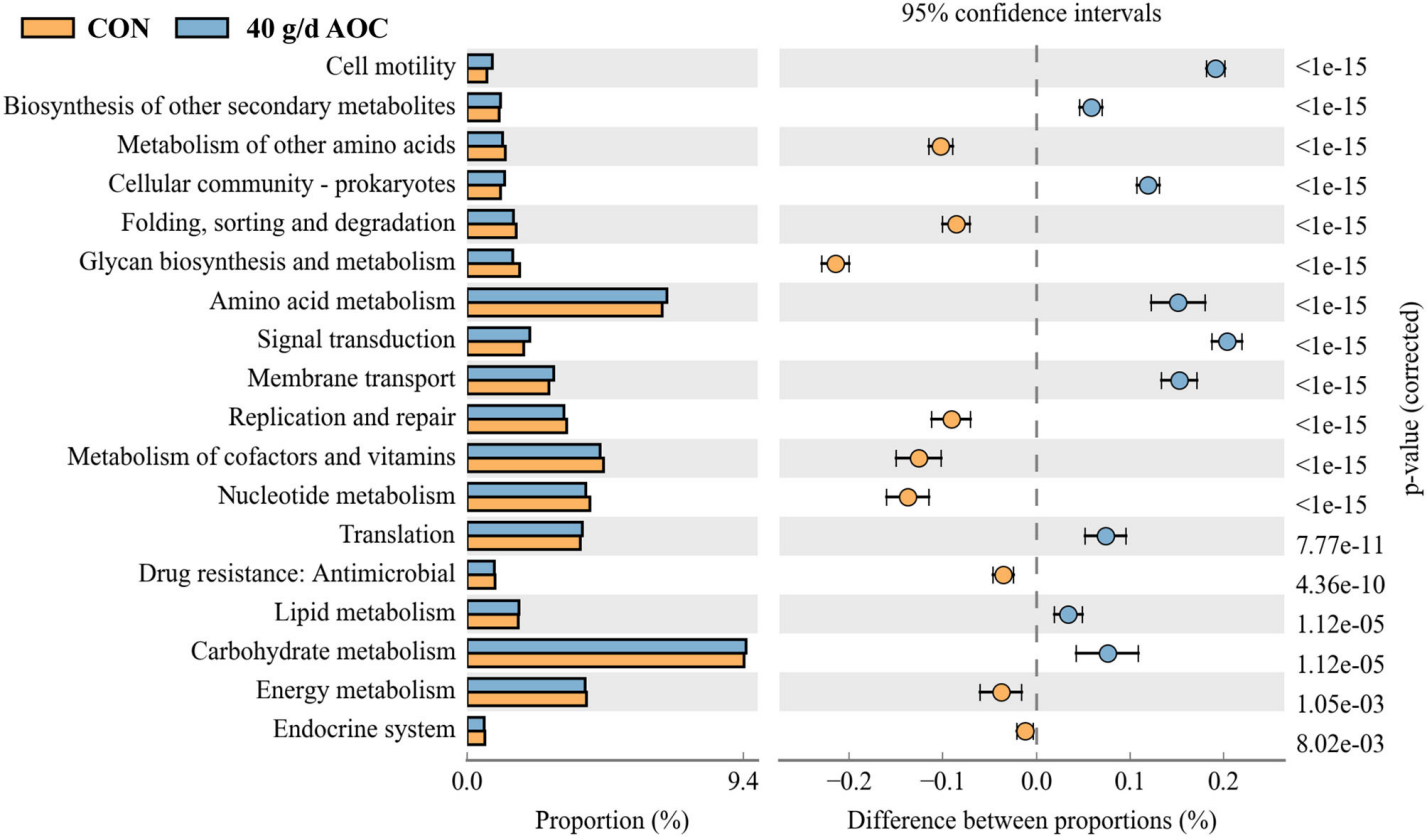
Predict of bacterial function in rumen by KEGG between CON and 40 g/d AOC supplementation using PICRUSt 2 method. Orange represent the CON, blue represent 40 g/d AOC treatment.

We also predicted the function of the bacterial functions in the rumen between the two treatments, which showed that the metabolism of carbohydrate, amino acid, and translation increased more while the metabolism of energy, cofactors and vitamins, and nucleotide decreased more in AOC treatment compared to those of CON treatment, respectively (Figure 4).

### Blood Cell

Compared with CON, AOC supplementation treatments did not significantly affect the composition of blood cells (*P* > 0.05, Table 8). The white blood cell (WBC) count tended to be lower in AOC treatments than in the CON treatment (*P* = 0.091). The blood cell composition of *Hu* sheep was in the normal range.

**Table 8:** Effects of different dietary levels of *Aspergillus oryzae* culture on blood cell count of *Hu* sheep.

**Items**	**Treatments**				**SEM**	***P*** **-value**		**R**
	**CON**	**AOC10**	**AOC20**	**AOC40**		**Linear**	**Quadric**	
RBC, M/μL	12.87	12.06	12.32	11.94	0.223	0.204	0.643	9.49~15.12
HCT, %	36.83	36.64	36.41	35.03	0.623	0.336	0.644	27.0~42.0
MCV, fL	29.71	30.04	29.20	29.50	0.294	0.588	0.984	24.4~32.5
MCH, pg	10.46	10.63	10.61	10.67	0.095	0.496	0.794	8.5~11.8
MCHC, g/dL	35.35	35.46	36.39	36.83	0.369	0.113	0.827	8.5~11.8
WBC, K/μL	9.96	9.84	7.91	8.10	0.378	0.023	0.827	5.06~14.12
NEUT, K/μL	3.54	3.88	2.34	2.99	0.259	0.161	0.766	1.17~6.11
LLYMPH, K/μL	3.77	3.99	4.24	3.71	0.153	0.413	0.566	2.54~9.60
MONO, K/μL	1.59	1.25	1.09	0.98	0.115	0.882	0.915	0.10~1.01
EO, K/μL	0.47	0.32	0.38	0.41	0.048	0.832	0.38	0.05~0.95
BASO, K/μL	0.01	0.02	0.04	0.02	0.006	0.504	0.146	0.00~0.12
HGB, g/dL	13.48	13.50	12.83	12.89	0.229	0.790	0.497	10.0~14.9
PLT, K/μL	406	401	450	390	17	0.997	0.461	301~922
MPV, fL	8.44	8.43	8.64	8.44	0.103	0.828	0.661	~
PCT, %	0.35	0.41	0.39	0.33	0.023	0.822	0.23	~

*CON, AOC10, AOC20, AOC40 are represent supplementation with Aspergillus oryzae culture 0 g/d, 10 g/d, 20 g/d, and 40 g/d, respectively; RBC, red blood count; HCT, hematocrit; MCV, mean corpuscular volume; MCH, mean corpuscular hemoglobin; MCHC, mean corpuscular hemoglobin concentration; WBC, white blood cell; NEUT, neutrophil count; LYMPH, lymphocyte count; MONO, mononucleosis; EO, eosinophilia; BASO, basophilic; HGB, hemoglobin; PLT, platelet count; MPV, mean platelet volume; PCT, platelet hematocrit; R, reference range of sheep blood routine indexes*.

## Discussion

In the present study, AOC supplementation increased the 24 h degradation rate of DM, and 40 g/d AOC increased the DM degradation rate of alfalfa hay and corn straw by 18.53% and 18.08%, respectively. The main reason for this is that AOC contains high activity enzyme series (23), including neutral protease activity (1020 IU/g), alkaline protease activity (770 IU/g), and cellulase activity (482 IU/g), so that it can significantly increase the activity of microcrystalline cellulase in alfalfa meal and corn straw, therefore accelerating fiber cracking (24) and microbial colonization in feed, and thus significantly increasing the DM degradation rate of alfalfa hay and corn straw at 24 h. Previous studies also obtained similar results. Newbold et al. (25) and Tricarico et al. (26) found that AO extract can increase the DM intake of lactating cows and heifers, and feeding 5 g/d of AO *in vitro* can improve the DM degradation rate of hay and barley in the artificial rumen for 24 h. Gomez-Alarcon et al. (9) conducted experiments on cows at dry and lactation stages and showed that feeding 3 g/d of AO could improve the fiber degradation rate and digestibility of the total digestive tract. In addition, Sun et al. (27) reported that AOC supplementation had a positive effect on rumen MCP in dairy cows, which may be due to the improvement in microbial activity. The results indicated that AOC could stabilize the rumen fermentation environment, ensure the growth of beneficial bacteria, increase the number of total anaerobic bacteria, and reduce the number of protozoa; therefore, more rumen bacteria can survive, which is conducive to the fermentation and degradation of nutrients in the rumen, thus improving the degradation rate of DM at 24 h. The predicted functions of rumen bacteria were also proved that the carbohydrate and lipid metabolism were improved in the AOC treatments, which indicated that the greater degradation rate of DM in the rumen could be advantageous for bacterial metabolism.

In the present study, feeding AOC significantly increased the content of TVFAs, including butyrate, butyrate, isovalerate, and valerate. Aditionally, the ratio of acetate to propionate decreased in the AOC-40 g/d treatment. Combined with the results of the DM degradation analysis, the increasing DM degradation in 24 h of alfalfa hay, corn stalk, and corn germ meal led to the greater TVFA content in the AOC supplementary treatments (20 g/d and 40 g/d). The same results have been reported previously: AOC could improve the rumen TVFA content, promote the decomposition of fiber, improve the rumen fermentation efficiency, and produce VFAs (5, 24). Gomezalarcon et al. (9) reported that TVFA content tended to be higher and that the molar ratio of A/P was lower (2.64 vs. 3.01) in high concentrate diets. Jouany et al. (28) found that feeding AOC could significantly reduce acetic acid content and increase propionic acid and butyric acid content. The unaffected acetate propionate concentration among treatments may be caused by the diet used in the present study. Isovalerate is a growth factor for some types of fibrolytic bacteria in the rumen. The increase in isovalerate may improve the microbial community structure to raise the DM degradation rate, which matched the results of the present experiment.

The TVFA affected the pH in the rumen after feeding. The dynamic change in the rumen pH was caused by the quick fermentation of the fast-degrading carbohydrates in the rumen. The rate of VFA production was quicker than the absorption of rumen epithelium and outflow rates; therefore, the accumulation of VFA decreased the pH. Afterward, the fermentation drops made VFA output balance with absorption, and the pH recovered to the normal range. The rumen pH value was not affected by AOC supplementation in the present study for either the mean, maximum, or minimum pH. These results were supported by Higginbotham et al. (6) and Fondevila et al. (23), who found that the AOC did not affect rumen pH in lactating dairy cows or sheep. However, some researchers reported that feeding 1 g/d, 2 g/d, 4 g/d, and 6 g/d AOC produced an effect, but without a clear trend for dose dependency in cattle (29), while feeding 27 g/d of AOC tended to lower the rumen pH (30). The different results were related to the different supplementation amounts of AOC, the basic diet (the ratio of concentrate to roughage), and ruminant species, all of which could affect the response of rumen pH to AOC supplementation. The dynamic trend of rumen pH after ingestion is to first decrease and then increase, returning to a normal physiological level. The ratio of concentrate to roughage in the diet used in the present study was 80 to 20, which is a typical diet for fattening sheep. The presence of a large amount of concentrate could make the rumen pH less affected by AOC. Rumen pH was mainly determined by the number of feeds converted into VFA yield by rumen microorganisms in the rumen after feeding. Interestingly, the dynamic changes of rumen pH in 24 h in the present study showed that the recovery of the pH value after intake was quicker in the AOC supplementation treatments than in the control treatment (from 5.6 to 6.4). This may be related to the greater DM degradation rate at 24 h in the AOC treatments, especially in the AOC-40g/d group. The rumen pH fluctuates within the range of 5.0 ~ 7.5, while the optimal pH of AO cellulase activity is 5.5 ~ 6.0. The pH after feeding AOC could provide a suitable acid-base environment for AOC cellulase to exert its maximum effect.

The α-diversity indices of all treatments were not significantly different in the present study. Combined with the results of rumen fermentation and pH, AOC did not affect the rumen bacterial diversity, which indicated that the rumen microbial flora maintains a better steady- state after AOC supplementation. Other study reported that the rumen bacterial community was changed after feeding with DFMs (31). The inconsistent results among studies were likely due to differences in diet treatments, dietary starch contents, and experimental animals.

Our results indicated that AOC significantly positively regulates the abundance of *Kiritimatiellaeota*. Although few data on sheep rumen have described *Kiritimatiellaeota*, these families and their classes are relevant to the metabolic body weight, dry matter intake, and residual feed intake of sheep (32). In addition, *Kiritimatiellaeota* has been reported to be saccharolytic and derive energy via fermentation (33). In the present study, AOC supplementation prompted an increase in *Kiritimatiellaeota* abundance, which may lead to an increase in DM digestibility and changes in rumen fermentation parameters, especially TVFA. Due to a lack of information on *Kiritimatiellaeota*, there was no significant difference at the genus level of rumen bacteria. The present study did not measure the DMI of the sheep because we used an *in situ* experimental design. Perhaps *Kiritimatiellaeota* in feeding regulation provides a new target in follow-up fattening experiments. In the present study, the AOC-40 g/d supplementation group and the CON treatment had different bacterial community structures in the rumen of *Hu* sheep. The bacterial community structure of various individuals was also different. The variation in the present study was the dosage of AOC. Due to the stability of the microbial community in the rumen (34), the results of β-diversity were also understandable.

Hematological parameters such as blood cell content are typically auxiliary indexes used to monitor health conditions or metabolic processes of animals (13). The blood cell analysis results of the sheep in the present study showed that all indicators were within the normal reference range (35), and AOC supplementation did not affect the blood cell composition, indicating that the experimental sheep remained healthy and free from disease. Previous studies have shown that AO and its culture have different effects on serum biochemical indices such as phosphorus, urea nitrogen, and alkaline phosphatase activity (36, 37), while the effect on blood cell composition has not been studied. The effects of nutritional additives on hematological parameters in sheep (13) and Nile tilapia (38) were usually insignificant, which were consistent with our results.

## Conclusion

The supplementation of *Hu* sheep with AOC could improve feed DM degradation and increase the energy supply of TVFAs in the rumen. Based on the feed conditions of the present study, supplementation 40 g/d of AOC could increase the production efficiency of sheep while higher level have to further investigate.

## Data Availability Statement

The data presented in the study are deposited in the NCBI repository, accession numbers SRR17532854, SRR17532869, and PRJNA795832.

## Ethics Statement

The animal study was reviewed and approved by the Animal Protection Committee of Gansu Province, China (2005-12).

## Author Contributions

LG and DZ collected the sample, analyzed the data, and drafted the manuscript. RD, FaL, and TR collected the sample. FeL presented the idea of this manuscript, supported the funding, analyzed the conclusions, and revised the manuscript. All authors contributed to the article and approved the submitted version.

## Funding

This research was financially supported by the Natural Science Foundation of Gansu (China), Grant Number 20JR10RA299.

## Conflict of Interest

The authors declare that the research was conducted in the absence of any commercial or financial relationships that could be construed as a potential conflict of interest.

## Publisher's Note

All claims expressed in this article are solely those of the authors and do not necessarily represent those of their affiliated organizations, or those of the publisher, the editors and the reviewers. Any product that may be evaluated in this article, or claim that may be made by its manufacturer, is not guaranteed or endorsed by the publisher.
